# Reconstruction of the crystalline lens full-geometry from OCT images acquired with off-axis viewing

**DOI:** 10.1038/s41598-026-42539-3

**Published:** 2026-05-11

**Authors:** Alvaro de la Peña, Javier Rodriguez-Sanchez, Estela Vadillo, Celia Talaván-González, Ester Carreño, Alberto de Castro, Susana Marcos, Eduardo Martinez-Enriquez

**Affiliations:** 1 Instituto de Óptica “Daza de Valdés”, Consejo Superior de Investigaciones Científicas (CSIC), Madrid, Spain; 2 Department of Epidemiology of Chronic Diseases, National Center for Epidemiology, Instituto de Salud Carlos III, Madrid, Spain; 3 Ophthalmology Department, La Paz University Hospital, Madrid, Spain; 4 Flaum Eye Institute, University of Rochester, Rochester, NY USA; 5 Center for Visual Science, University of Rochester, Rochester, NY USA

**Keywords:** Eye, Crystalline lens geometry, Aging, Presbyopia, Cataract, Optical Coherence Tomography, Diseases, Medical research, Optics and photonics

## Abstract

The crystalline lens of the eye is an optical structure that, together with the cornea, focuses images onto the retina. In a young and healthy eye, the lens is transparent and adjusts its shape to focus on near and distant objects (accommodation). With age, it loses this flexibility (presbyopia) and may later become opaque (cataract). Understanding the full three-dimensional geometry of the crystalline lens is essential for studying age-related lens growth and accommodation mechanism, as well as for the customized design and selection of intraocular lenses the for implantation in cataract surgery. While Optical Coherence Tomography (OCT) allows imaging of the central lens region visible through the pupil, conventional in vivo measurements cannot capture the full lens shape, particularly at the periphery, due to iris obstruction. In this study, we present a novel method to reconstruct the full three-dimensional shape of the crystalline lens by combining OCT images acquired with off-axis illumination at different angles. Using this approach, full lens reconstructions were achieved in eight subjects, and key geometrical parameters - diameter, volume, and surface area- were quantified, showing strong positive correlations with age. Comparisons with estimates derived from pupil-limited regions showed strong agreement, thereby validating these estimation methods.

**Supplementary Information:**

The online version contains supplementary material available at 10.1038/s41598-026-42539-3.

## Introduction

Quantifying the full shape of the crystalline lens in the human eye plays an important role in numerous clinical and surgical applications. These include cataract surgery planning, such as intraocular lens (IOL) selection or sizing of accommodating IOLs, and the design of next-generation of IOLs^1^. Furthermore, accurate reconstruction of the lens’s full three-dimensional (3-D) morphology is essential for understanding ocular growth in children, including the role of crystalline lens remodeling in emmetropization, and how its failure can lead to myopia^2,3^. Accurate lens geometrical models are therefore of interest in the understanding and development of correcting alternatives for myopia and presbyopia. They also provide the geometrical information needed for biomechanical modeling of the lens for studying accommodation^4^.

Optical Coherence Tomography (OCT)^5^ is commonly used to capture 3-D images of the eye due to its advantageous trade-off between speed and resolution and its availability in clinical settings. Nevertheless, it provides access only to the limited central portion of the lens visible through the pupil because the iris blocks the incident light, preventing obtaining information of the periphery of the lens. Some imaging modalities have been employed to assess the full geometry of the crystalline lens, including its peripheral regions. Magnetic Resonance Imaging (MRI) and Ultrasound Biomicroscopy (UBM) have demonstrated the capability to visualize the entire lens structure^6–10^. However, MRI is limited by relatively low spatial resolution (~ 100 μm), prolonged acquisition times (several minutes), high cost, and limited clinical availability. UBM offers improved resolution (~ 40 μm) and faster imaging (milliseconds), but requires contact with the ocular surface and a skilled operator, implying patient discomfort and clinical constraints.

In absence of images of the peripheral lens geometry with OCT, the *intersection approach* has been proposed to estimate the lens diameter from the portion of the lens visible through the pupil. This method extrapolates the lens periphery by fitting two parametric surfaces (usually circles) to the visible anterior and posterior lens surfaces and determines the equatorial diameter from their intersection. The intersection approach has been applied in several studies^11–14^ and is implemented in commercial anterior segment OCT systems, including CASIA2 (Tomey, Nürenberg, Germany) and Catalys femtosecond laser platform (Johnson & Johnson Vision, Santa Ana, CA, USA). However, previous investigations on ex-vivo lenses measured with OCT, in which the full geometry of the lens is visible because the iris is removed, demonstrated that the intersection approach introduces systematic errors, notably overestimating both the equatorial diameter and lens volume (by 15% and 11% on average respectively), while underestimating the position of the equatorial plane by shifting it anteriorly (by 17%)^15^.

In previous studies, we proposed two methods to accurately estimate the full shape of the crystalline lens from its visible central region^15,16^. Both methods rely on geometric models trained with ex-vivo OCT data of complete lens geometries. One approach uses parametric descriptions of different regions of the lens^15^, while the other applies a basis decomposition into *eigenlenses*. In this latter approach, each lens shape is represented as a weighted sum of a small set of *eigenlenses*, with the most significant *eigenlenses* capturing the dominant deformation patterns across a training set of lenses^16^. These full-shape estimation methods have been applied to study changes in lens geometry with accommodation^17,18^ and with refractive error^17,19^. The accuracy of the *eigenlens* representation has been validated in isolated ex vivo lenses^16^ and in lenses mounted on a lens stretcher that simulate physiological accommodation^20^. In addition, their ability to capture in vivo shape changes with accommodation has been supported indirectly through comparisons with state-of-the-art MRI measurements^17^. However, a direct in vivo validation of these methods is still lacking.

In this study, we used off-axis imaging to access the peripheral regions of the crystalline lens. A previous study used oblique OCT scanning with five volumetric image acquisitions to enlarge the visible portion of the posterior crystalline lens surface^21^, achieving a 17% increase in volumetric lens coverage. However, that work neither imaged the peripheral regions nor reconstructed the true 3-D geometry of the lens. Previous works have proposed methods for stitching multiple 3-D OCT volumes, but they were not developed for in vivo crystalline lens imaging^22–25^.

The current study presents two key advances in the imaging and reconstruction of the human crystalline lens using OCT. First, we present, to the best of our knowledge, the first OCT images that capture the equatorial region of the crystalline lens. These images extend beyond the central optical zone typically visualized in previous studies with both laboratory-developed and commercial OCT systems, as well as beyond earlier attempts to enlarge the visible portion of the posterior lens surface^21^. Second, we demonstrate a complete 3-D reconstruction and quantification of the full lens geometry directly from the OCT data, with minimal reliance on extrapolation, unlike previous estimation methods^11,12,15–17^. This study thus represents the first in vivo validation of prior lens full geometry estimation methods, thereby demonstrating their clinical utility.

To image the peripheral regions of the crystalline lens, OCT scans were acquired at multiple fixation angles, resulting in off-axis illumination. Measurements from different angles revealed different regions of the posterior lens surface and extended coverage to the equatorial region of the lens. A complete 3-D reconstruction of the lens geometry was then achieved by registering all volumetric datasets within a common coordinate system.

This study reports reconstructions of the full crystalline lens shape in eight eyes and quantifies several geometric parameters, including lens volume, diameter, and surface area. In addition, we compare the proposed method with previously published estimation models^16,17^, based on *eigenlenses*.

## Methods

### Subjects

Measurements were acquired on eight eyes from eight subjects with no ocular pathology or surgery, with a mean ± standard deviation age of 29 ± 6 y/o (range: 23 to 40 y/o) and a mean refractive error of −1.6 ± 2.9 D (range: −6.75 D to + 2 D). Measurements were performed under pharmacologically induced mydriasis, by one drop of tropicamide 10 mg/ml and phenylephrine 100 mg/ml to dilate the pupil. The subjects were instructed to wait 25 min to ensure adequate onset of phenylephrine’s pharmacologic effect. The study was conducted in accordance to the Declaration of Helsinki. The study was approved by Institutional Review Board “Comité de Ética del CSIC”. All participants in the study provided signed informed consent forms. All participants in this study provided consent for publication of their image.

### OCT system

We acquired the OCT images using the commercial OCT system ANTERION (Heidelberg Engineering, Heidelberg, Germany) that uses a swept-source laser with a wavelength of 1300 nm at a speed of 50,000 A-scans/s. The axial resolution is < 10 μm in tissue. It includes an integrated infrared (IR) camera and an active eye tracking mechanism to ensure stable and precise image acquisition. The ANTERION system includes four different measurement modalities: Cornea App, Cataract App, Metrics App, and Imaging App. In this work, we used the Imaging App to obtain images with personalized radial scanning consisting of 65 meridional B-scans (meridians) with a 14 mm lateral scan range each and 256 A-scans/B-scan. We chose these parameters to achieve a good trade-off between spatial resolution and total acquisition time of a 3-D data set. The images obtained from the Imaging App are raw images in pixels, which must be converted to millimeters using calibration information as explained below. More details on the ANTERION OCT system can be found in published reports^26^.

### Data acquisition

We imaged one eye per subject (OS in 7 subjects and OD in 1 subject). The eye was imaged with light incident from 8 different orientations (i.e., obtaining 8 volumetric 3-D measurements, one for each orientation), using an external stimulus in order to measure the periphery of the lens at different areas. These orientations were: on axis (OCT beam centered, i.e., typical OCT measurements); subject gazing nasally at 30º and 45º; subject gazing temporally at 30º and 45º; subject gazing superiorly at 20º and 30º; and subject gazing inferiorly, at 45º. These angles are approximate values estimated directly from the relative position between the stimulus and the instrument axis, and were not measured with high precision. Nevertheless, they were selected to introduce redundancy in the acquired data; therefore, this approximation does not affect the reconstruction (see Discussion).

**Fig. 1:**
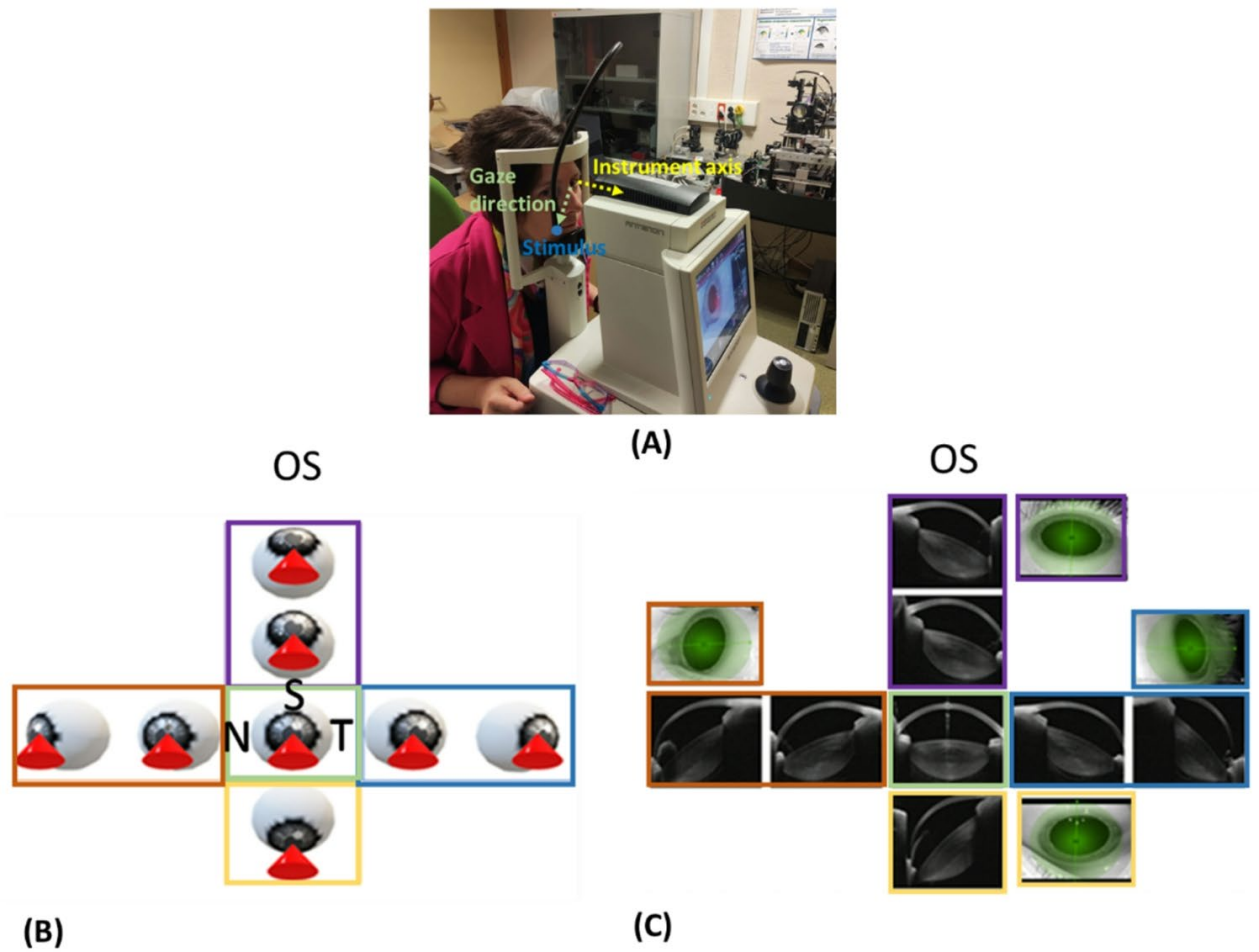
OCT imaging using lateral incidence at 8 different eye orientations. (**A**) Measurement setup, showing the external stimulus, the gaze direction and the instrument axis. (**B**) Illustration of the 8 different fixations for OCT acquisition. The orientation of the eye illustrates the eye rotation with fixation. The red cone indicates the beam incidence. N, S and T stand for Nasal, Superior and Temporal, respectively. (**C**) Example of a representative OCT meridian (extracted from each volumetric scan) and pupil camera image for each corresponding orientation. For clarity, pupil camera images are presented only at higher lateral incidence angles: 45° nasally, temporally, and inferiorly, and 30° superiorly. Colored lines in boxes indicate incidence: Green line: normal incidence; Red: nasal incidence (30 and 45 deg.); Blue: temporal incidence (30 and 45 deg.); Purple: superior incidence (20 and 30 deg.); Yellow: inferior incidence (45 deg.).

Figure 1A illustrates the measurement setup. Figure 1B presents a schematic of the different orientation angles measured. Figure 1C shows an example of one of the meridians obtained (extracted from each volumetric scan) and the corresponding pupil camera image for each orientation. The complete measurement session for a single eye lasted approximately 20 min following pupil dilation, and it comprised the change in fixation target and acquisition of a volumetric set per fixation condition (i.e., light orientation). Images containing artifacts (e.g., motion, eyelid interference) were excluded. To evaluate repeatability, three volumetric datasets were acquired for each fixation condition in two subjects.

### Methodology to obtain the full shape of the crystalline lens

**Fig. 2:**
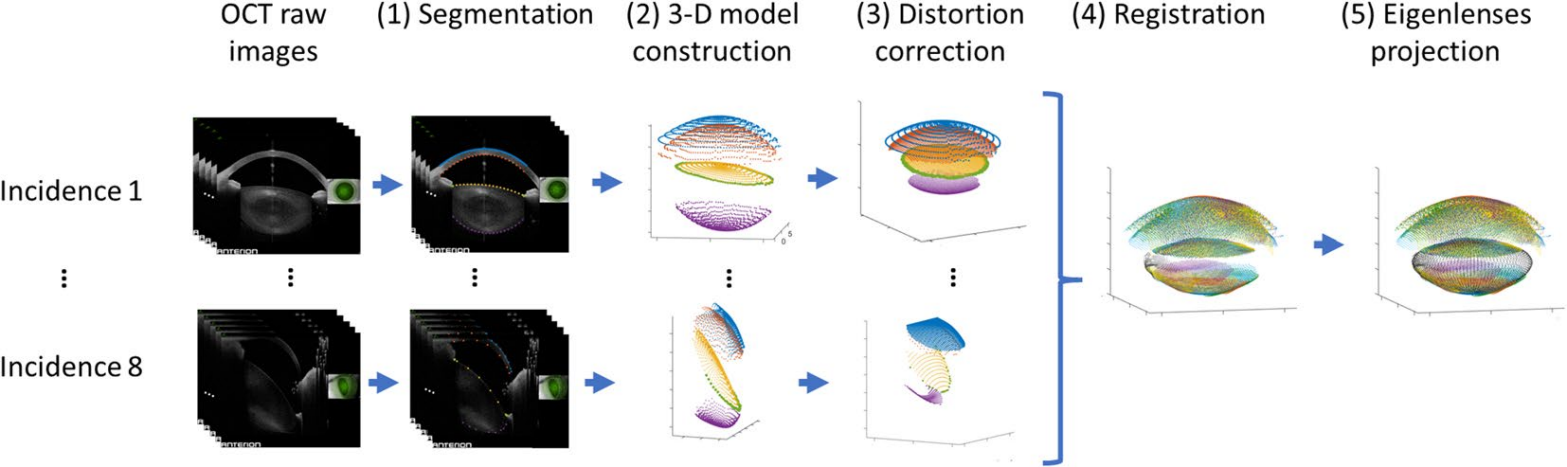
Main process involved in the construction of the full shape of the crystalline lens.

Figure 2 illustrates the algorithm developed to obtain the 3-D full shape of the crystalline lens. It includes five steps: (1) OCT images segmentation; (2) 3-D model reconstruction for each incidence angle; (3) distortion correction; (4) registration of the 3-D models from different incidence angles; and (5) *eigenlenses* projection.

#### OCT images segmentation

The segmentation process involves identifying the anatomical surfaces of interest within the OCT images and classifying them as the anterior corneal surface, posterior corneal surface, anterior lens surface, posterior lens surface, and iris.

For normal and low incidence angles, an algorithm based on previous publications^18,27^ was developed to automatically segment each OCT image. For this study, we introduced several specific improvements to enhance segmentation performance, including: (i) the use of iris segmentation to restrict the processing area to the pupil, thereby improving the accuracy of crystalline lens segmentation; and (ii) the removal of eyelash artifacts in anterior cornea segmentation through an iterative process. In this process, the detected edges were first fitted to a low-order polynomial, and samples located beyond a predefined distance of 3 standard deviations from the fitted polynomial were discarded.

As the automatic algorithms failed at high incidence angles, we implemented a manual segmentation approach for those cases. Specifically, for each anatomical surface of interest in every OCT image, the operator selected at least five points by manually clicking with the mouse along the visible boundary. The manual procedure followed several guidelines: (i) the operator segmented the iris by selecting the most internal points on the “left” and “right” parts of the pupil (see green points in Supplementary Fig. S1); (ii) to avoid losing information in subsequent processing, the operators chose points to sample all visible regions of each surface. Remarkably, if, for example, a large portion of the anterior lens surface was visible but not “overlaid” by the cornea (i.e., the segmentation domain was lower in the cornea than in the anterior lens), the uncovered region of the lens would be lost during the distortion correction process; (iii) in most cases, the first and last points selected on the anterior lens corresponded to the iris segmentation.

Although time-consuming, this method provided reliable and consistent segmentation results across multiple orientations and subjects. Furthermore, small imperfections in the manual segmentation were effectively minimized as a surface smoothing was performed in the 3-D model reconstruction process (see next subsection).

All segmentation results were visually inspected, and the operator repeated the segmentation whenever the outcome was deemed unsatisfactory (e.g., in images with low signal to noise ratio, the automatic algorithm produced obvious deviations, particularly at the lens surfaces). Importantly, segmentation was performed by four independent operators and evaluated by a highly experienced operator, who assessed the impact of different segmentations on the quantification results^28^. Segmentation results were consistent across operators, showing no significant differences.

Supplementary Fig. S1 shows segmentation examples on specific meridians of 3 different incidence angles for the same subject.

#### 3-D model reconstruction

After segmentation of all the images, the 3-D geometry was reconstructed for each of the eight different incidence angles. We converted the coordinates of the segmented surfaces from pixels to millimeters (9.2 μm/pixel), using calibration data obtained from measurements of a reference grid and calibration spheres with known radius of curvature. Then, we registered the meridians into a common 3-D space by mapping the segmented surfaces according to their corresponding scanning angle (from 0 to π, with angular sampling at intervals of 0.048 rad, i.e., 2.7 º) and direction (i.e., where the scan starts and ends, e.g., from the nasal-superior quadrant to the temporal-inferior quadrant). Finally, we approximated the ocular surfaces using Zernike polynomials^29,30^, providing smooth and uniformly sampled 3-D geometries. Specifically, we used 15 Zernike terms, and determined the coefficients by fitting each surface using least-squares.

**Fig. 3:**
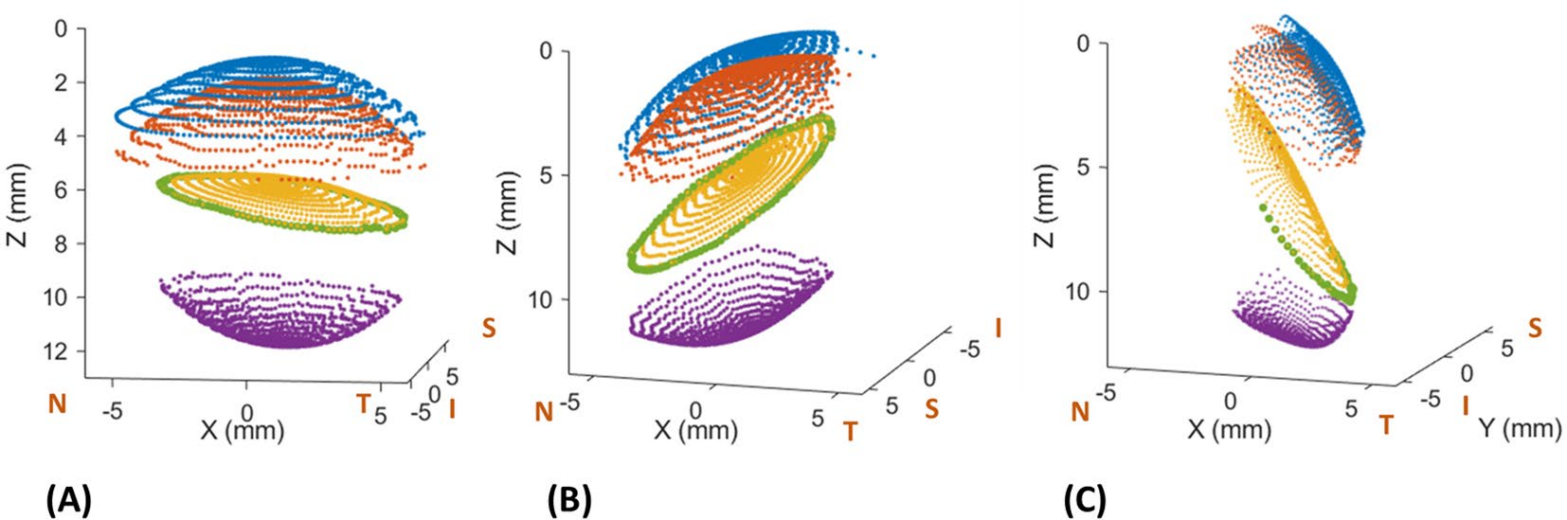
3D model reconstruction. (**A**) Normal incidence; (**B**) nasal 30 degrees incidence; (**C**) temporal 45 degrees incidence. N, S, T and I stand for nasal, superior, temporal and inferior respectively. The blue color indicates anterior cornea surface; red, posterior cornea surface; yellow, anterior lens surface; purple, posterior lens surface; and green indicates the iris. Examples are for Subject#1 (OS).

Figure 3 illustrates the volumetric models obtained for three different incidence angles for subject #1 (OS), namely, normal incidence, nasal (30 degrees) incidence, and temporal (45 degrees) incidence.

#### Optical distortion correction

The aim of optical distortion correction is to recover the true geometry of ocular structures by compensating for the geometric distortions introduced by refractive elements in the anterior segment of the eye. In particular, when imaging the posterior surface of the lens, light rays refract at the cornea and the anterior surface of the lens, altering both their propagation direction and optical path length due to surface curvature and refractive index differences^31^.

To correct for these effects, we exported the anterior corneal surface data into an optical design and simulation program (Zemax, Ansys, PA), traced the rays corresponding to the OCT sampling region, and determined their intersections with the anterior corneal surface. We computed the refracted direction vectors of these rays and, for the reconstruction of the posterior corneal surface, traced each ray along its refracted path for a distance equal to the optical path length (measured between the anterior and posterior corneal surfaces in the OCT images), divided by the group refractive index of the cornea. We applied an analogous iterative reconstruction procedure to reconstruct the crystalline lens surface. Specifically, the anterior lens surface was reconstructed first, followed by the posterior surface, ensuring that the influence of refraction at each preceding boundary was accounted for in the subsequent step. Supplementary Fig. S2 illustrates the optical distortion correction process, showing the uncorrected and corrected surfaces for subject #1. As will be further elaborated in the discussion section, it is essential to perform this correction using three-dimensional ray-tracing algorithms rather than applying independent two-dimensional corrections to each meridian, as is commonly implemented in certain commercial OCT systems. The group refractive indices used in the reconstruction were 1.385 for the cornea^32^, 1.345 for the aqueous and 1.417 for the crystalline lens^33,34^.

#### Registration of 3-D measurements acquired from different incidence angles

The eight 3-D models reconstructed from different incidence angles, each representing a distinct portion of the crystalline lens, were subsequently registered within a common coordinate system to generate the final geometry of the complete lens. To this end, we rotated all 3-D models and translated them such that the iris was aligned across all incidence angles. We chose the iris as the reference structure because it was visible in nearly all meridians across measurements. Specifically, we approximated the segmented iris by a plane, and used the normal vector to this plane to correct the tilt by applying a rotational transformation. Subsequently, we fitted the iris points to a circle, and calculated the displacement vector along the X, Y, and Z axes required to place the circle center at the coordinate origin (0,0,0). We then applied the resulting rotation matrices and displacement vectors to all reconstructed surfaces.

**Fig. 4:**
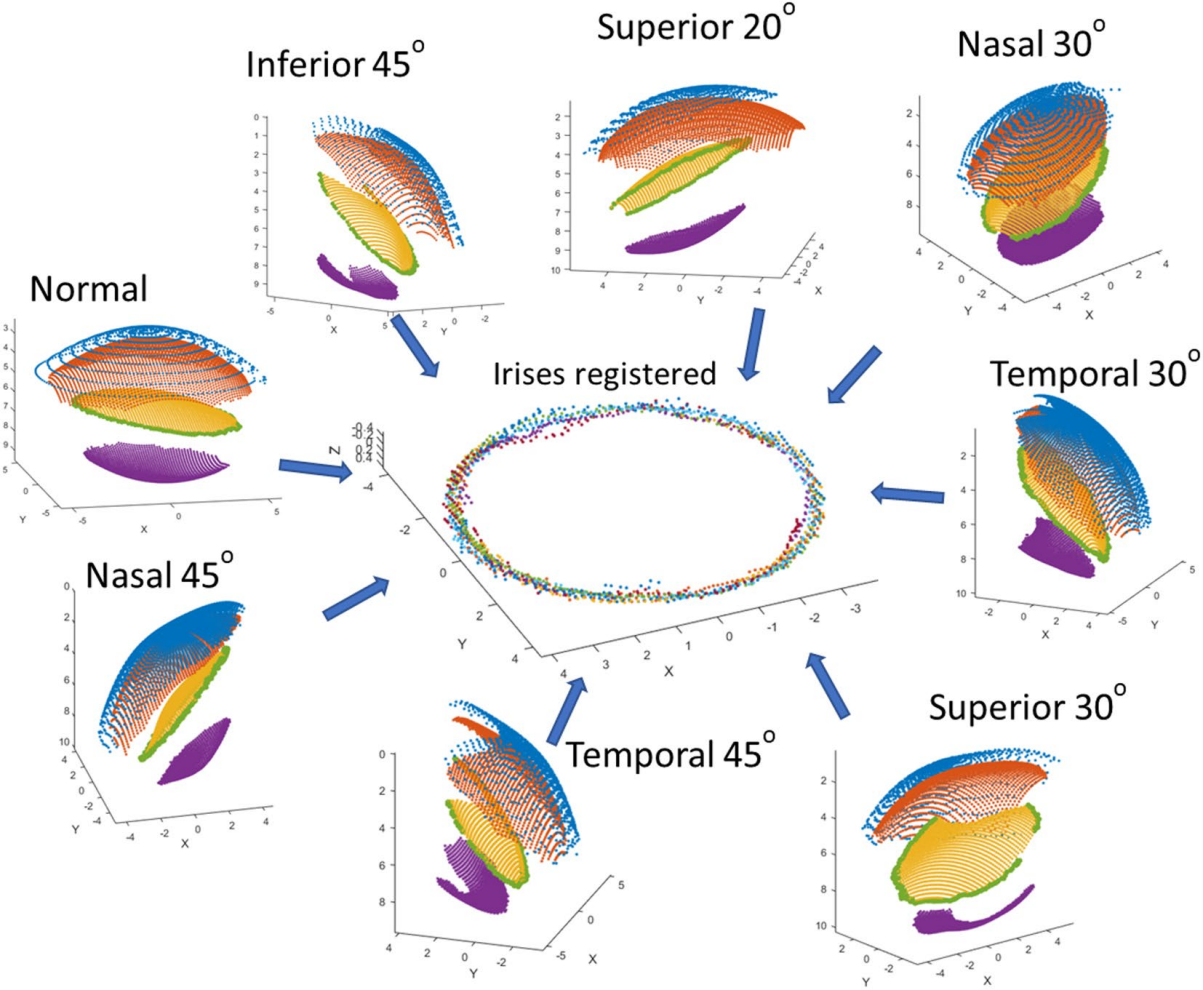
Registration process of the eight 3-D models from the measurements at different incidence angles and their corresponding Iris data after registration. Examples are for subject #1 (OS).

Figure 4 shows the eight 3-D models from the measurements at different incidence angles and in the center of the figure the eight irises in the same coordinate system (after the registration). Supplementary Video S3 shows how this process is performed dynamically.

**Fig. 5:**
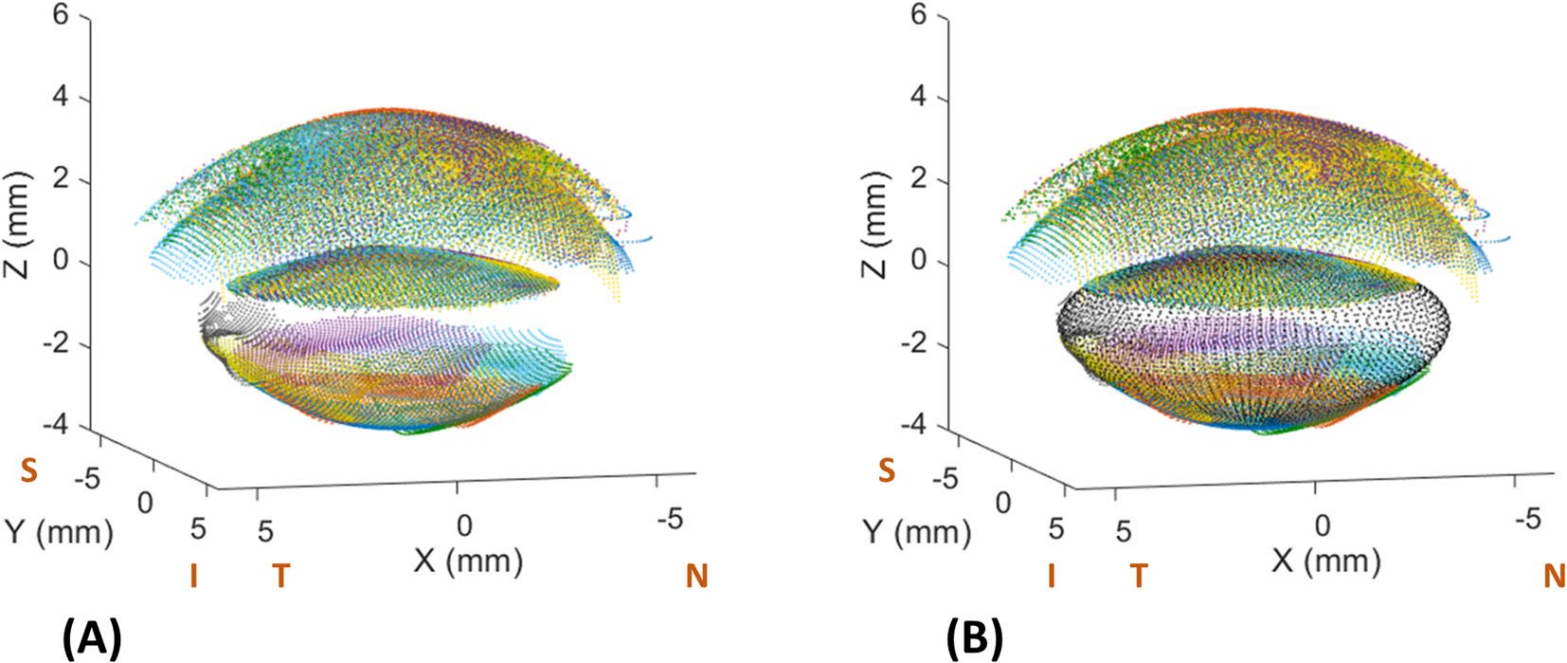
Registered models, before and after fitting to eigenlens basis. (**A**) Combination of data for 8 incidences after the registration process; (**B**) Data from (**A**), with eigenlens fitting superposition (in black). Dark blue: Normal incidence; Red: Inferior incidence; Yellow: Superior 30 deg. incidence; Purple: Superior 45 deg. incidence; Green: Nasal 30 deg. incidence; Blue: Nasal 45 deg. incidence; Olive: Temporal 30 deg. incidence; Charcoal: Temporal 45 deg. incidence. See supplementary video S4 to see the registration process step by step. N, S, T and I stand for Nasal, Superior, Temporal and Inferior respectively.

Following initial registration, we refined the alignment using an iterative closest point (ICP) algorithm^35^ to optimize the rigid transformation between the eight 3-D models. This algorithm minimized the mean squared error between the point sets by iteratively updating rotation and translation parameters. This step is a refinement to correct the minimum errors that may have occurred in the rotations and translations using the iris as a reference, especially in the most extreme incidence angles. Figure 5A shows the eight incidences after the registration process, where each color represents the 3-D model of each incidence.

Supplementary video S4 shows the registration of the different incidences dynamically.

#### Smoothing the full shape crystalline lens using eigenlenses

Finally, we projected the reconstructed and registered 3-D model of the crystalline lens full shape onto a base of *eigenlenses*. This step is very relevant for three important reasons: (i) to obtain a compact representation of the lens shape, useful in applications as intraocular lens position and tilt estimation in cataract surgery^36,37^; (ii) to smooth the reconstructed surface, filtering out noise and local irregularities that may result from imaging artifacts or registration inaccuracies; and importantly (iii) to obtain the lens shape in the full domain, including some areas that were not completely visible in the OCT images (i.e., obtain a closed form shape of the lens). This is further discussed in the Results and Discussion sections. Specifically:

1. The 3-D crystalline lens full-shape dataset were first aligned by centering laterally at the anterior lens maximum elevation point and axially at the midpoint between the anterior and posterior surfaces. The aligned data was then transformed into polar coordinates and interpolated onto a fixed sampling grid consisting of P = 100 equidistant elevation angles and Q = 100 equidistant azimuth angles. This grid defines the lens surface with M = P×Q = 100 × 100 = 10,000 points, from which the surface elevation values$$\begin{aligned} \:\bf{l} \end{aligned}$$ (defined as the radial distance from the origin of coordinates) were obtained.
2. The residual data was obtained by subtracting the mean lens$$\begin{aligned} \:\mathbf {\bar{l}} \end{aligned}$$ (already calculated and saved as a vector from the training set as indicated in)^16,17^, and projected into a basis of six *eigenlenses*:    

$$\bf{r} = \bf {l} - \bf {\bar{l}},$$

$$\mathbf{a} = ( a_1, \dots, a_6 )^{\mathrm{T}} = \mathbf{M}^{\mathrm{T}} \mathbf{r}= ( \mathbf{e_1} \mathbf{r}, \dots, \mathbf{e_6} \mathbf{r} )^{\mathrm{T}}, $$

where $\it{ \bf {a}}$ is a vector with the 6 coefficients, $\mathbf{a} = (a_1, \dots, a_6)^{\mathrm{T}}$, that represents the shape of the lens in an *eigenlens* basis; $\mathbf{M}$ is an M×6 matrix where each column is the ordered (starting from the highest eigenvalue) first 6 *eigenlenses* $\mathbf{M}={\left({\mathbf{e}}_{1},\dots,{\mathbf{e}}_{6}\right)}^{};$ and$$\begin{aligned} \:\bf{l} \end{aligned}$$ is an M×1 vector of the data; T indicates the transpose operator, and bold indicates vectors and matrices. Six *eigenlenses* were chosen as they provided an optimal trade-off between accuracy and compactness^16^.

(3) Lens reconstruction: Once the vector of *eigenlenses* coefficients that defines the lens shape $\bf {a}$ have been obtained, the full shape$$\begin{aligned} \:\bf{\hat{l}} \end{aligned}$$ is estimated by projecting into the *eigenlenses* basis and adding the mean lens$$\begin{aligned} \:\mathbf {\bar{l}} \end{aligned}$$:    

$$\hat{\mathbf{l}}=\mathbf{\bar{l}}+\sum_{k=1}^{K=6}{a}_{k}{\mathbf{e}}_{k}.$$

This projection ensures that the final lens shape adheres to realistic anatomical variations observed in the training population, improving the robustness of the reconstruction. Figure 5B shows the *eigenlenses* projection (in black) superimposed to the data after the registration process.

### Quantification

Once the 3-D models were constructed, we quantified various geometrical parameters from the crystalline lens full shape, specifically: (1) lens equatorial diameter (DIA); (2) lens surface area (LSA); and (3) lens volume (VOL). The LSA was estimated as the sum of the triangles formed by Delaunay triangulation; the VOL was estimated, using double integration over the region where the lens is defined, as the sum of the VOL of the anterior and the posterior parts of the lens^17,18^.

### Data analysis

We assessed the normality of the data using the Shapiro-Wilk test. Since lens volume did not meet the assumption of normality, non-parametric analyses were performed. Specifically, Spearman’s rank correlation coefficient was used to evaluate the association between the geometrical parameters of the crystalline lens and age, obtaining the Spearman correlation coefficient (ρ) and the p-value for testing the null hypothesis of no correlation (p). Bland Altman plots and linear regression analysis were used to assess agreement between the proposed method and previously reported crystalline lens full shape estimation methods from the pupil information^16,17^. The coefficient of variation (CV) was used to evaluate repeatability. For all analyses, statistical significance was defined as a p-value lower than 0.05. Calculations were obtained using MATLAB software (MathWorks, Natick, MA, USA).

## Results

### OCT images showing the crystalline lens periphery and full lens shape reconstruction

**Fig. 6:**
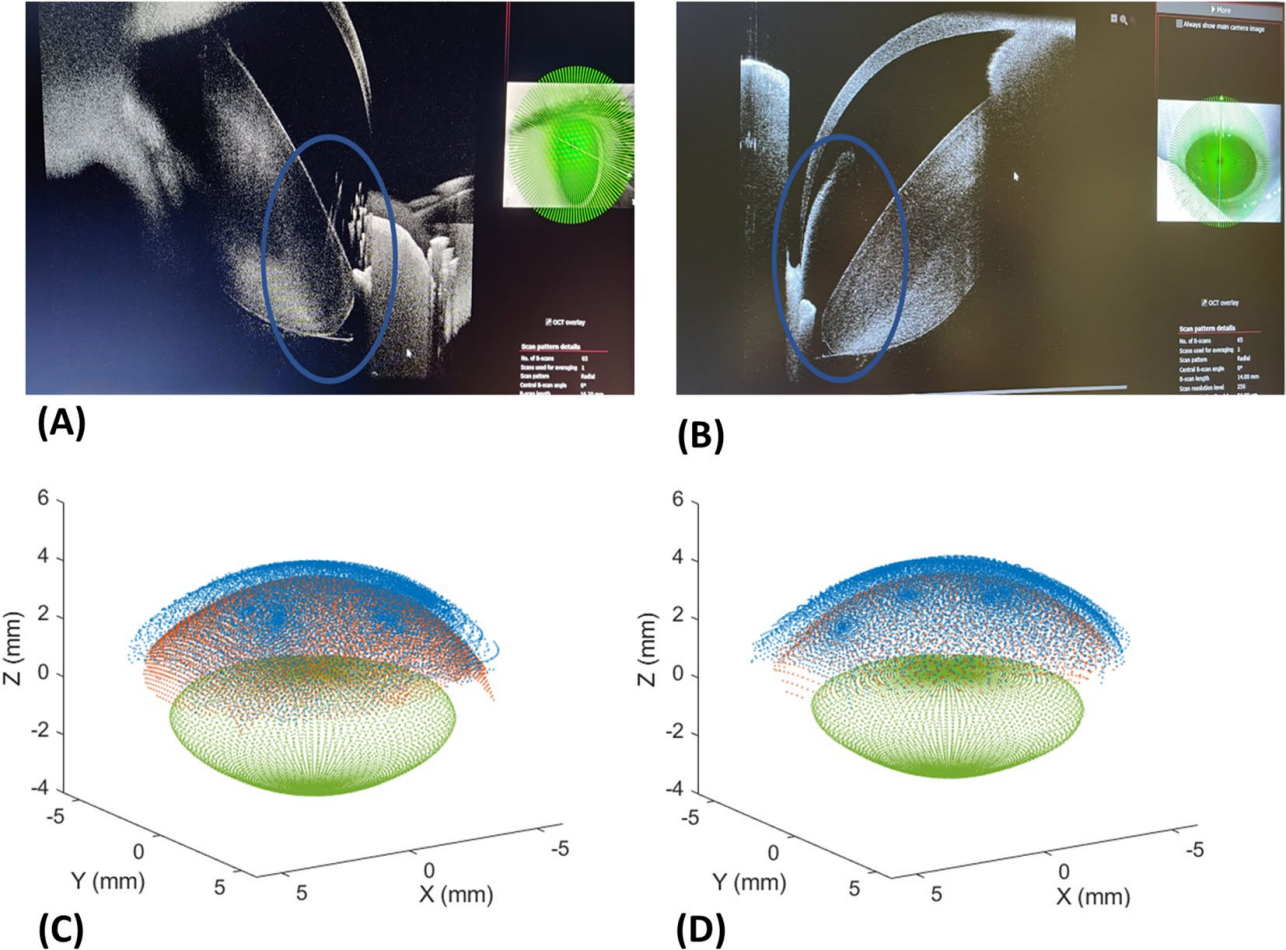
OCT raw images captured using lateral incidence (upper panels) and reconstructed 3-D geometry (lower panels). Blue ellipses in upper panels highlight the periphery of the crystalline lens, visible only under lateral incidence. (**A**) Subject #1, age 40 y/o, OS gazing temporally. (**B**) Subject #6, 26 y/o, OS gazing inferiorly. Surfaces in lower panels are represented by: blue for anterior surface; red for posterior surface; and green for the crystalline lens. (**C**) Subject #1. (**D**) Subject #6.

Figure 6 (upper panels) presents examples of raw OCT images of the crystalline lens periphery, acquired using the measurement protocol described above. Specifically, images are extracted from the volumetric measurements in two subjects: (A) Subject #1, 40 y/o, OS gazing temporally; and (B) Subject #6, 26 y/o, OS gazing inferiorly. Figure 6 (lower panels) shows the corresponding full 3-D reconstruction for the same subjects.

### Repeatability

To assess the repeatability of the method for measuring the full lens shape, we obtained three consecutive measurements for each incidence angle in two subjects (Subject #1 and Subject #3). Table 1 summarizes the corresponding DIA, VOL, and LSA values.

**Table 1:** DIA (mm), VOL (mm^3^) and LSA (mm^2^) obtained for three repeated measurements in two subjects.

		DIA (mm)	VOL (mm^3^)	LSA (mm^2^)
Subject #1	Measurement #1	9.45	188	178
	Measurement #2	9.48	190	179
	Measurement #3	9.55	191	181
Subject #3	Measurement #1	8.93	135	152
	Measurement #2	8.88	134	150
	Measurement #3	8.94	136	152

The coefficients of variation (CV) for Subject #1 were 0.54% for DIA, 0.85% for LSA and 0.80% for VOL, while for Subject #3, the CV values were 0.36% for DIA, 0.76% for LSA, and 0.74% for VOL. These low CV values (all below 1%) demonstrate high repeatability and consistency across repeated measurements for both subjects.

### Validation of estimation methods: comparison between full shape reconstruction from all projections and full shape estimation from the central region using eigenlenses.

We compared the lens geometric parameters (volume, equatorial diameter and lens surface area) obtained with the proposed method using lateral light incidence to those derived from the *eigenlenses* estimation method^16,17^, which reconstruct the full lens shape from the limited central region visible through the pupil under normal light incidence (standard OCT measurements commonly used in clinical imaging). This comparison allowed to evaluate the accuracy and reliability of estimation techniques that rely on partial data when the full shape of the lens is not available. Supplementary Fig. S5 compares the geometry accessible through the pupil with that obtained using the proposed method.

**Fig. 7:**
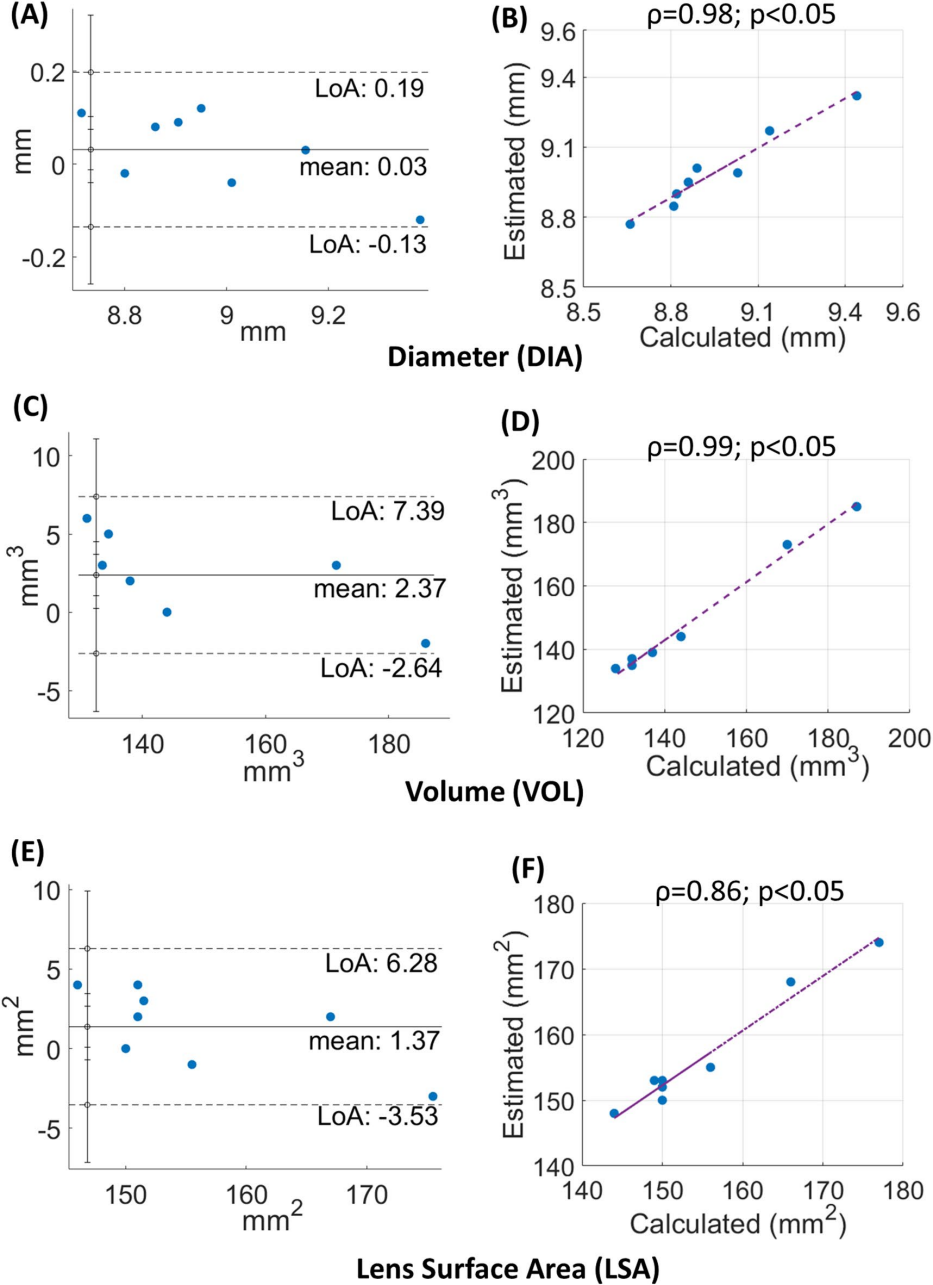
Comparison between the proposed method using all orientations and the estimation methods that estimate the full shape of the lens from the limited central zone (pupil, on-axis data) using *eigenlenses*^16,17^. (**A**) Diameter, Bland-Altman plot; (**B**) Diameter, correlation plot; (**C**) Volume, Bland-Altman plot; (**D**) Volume, correlation plot; (**E**) Lens surface area, Bland-Altman plot; (**F**) Lens surface area, correlation plot. In the correlation plots, “Calculated” (X-axis) refers to the values obtained with the proposed method, and “estimated” (Y-axis) refers to the values obtained with estimation methods from the pupil. Spearman correlation coefficient (ρ), p-values for testing the null hypothesis of no correlation (p), and best linear regression lines (purple dashed lines) are shown.

Figure 7 shows Bland-Altman (first column) and correlation (second column) plots for the lens DIA (first row) and VOL (second row). In Bland-Altman plots, the Y axis represents the mean difference (MD) between the methods calculated as the eigenlens estimation method minus the proposed method from lateral indicence. The limits of agreement (LoA) are calculated as 1.96·SD, where SD is the standard deviation of the difference. In the correlation plots, the term “calculated” (X-axis) refers to the values obtained with the proposed method, and “estimated” (Y-axis) refers to the values obtained with estimation methods from the pupil. Supplementary Fig. S6 shows Bland-Altman (first column) and correlation (second column) plots for the first two eigenlenses coefficients, ${a}_{1}$ and ${a}_{2}$, which to a large extent define the shape of the crystalline lens, explaining 80% of the variance^16^.

The Bland-Altman plots show a small MD and narrow LoA for DIA (MD = 0.03 mm; LoA: − 0.13 to 0.19 mm), VOL (MD = 2.37 mm^3^; LoA: − 2.64 to 7.39 mm³), LSA (MD = 1.37 mm^2^; LoA: − 3.53 to 6.28 mm^2^), ${a}_{1}$ (MD=−0.4; LoA: −5.09 to 4.29) and ${a}_{2}$ (MD=−1.47; LoA: −4.64 to 1.69). The correlation plots show a high correlation between estimated and calculated values for DIA (ρ = 0.98), VOL (ρ = 0.99), LSA (ρ = 0.86), ${a}_{1}$(ρ = 0.99) and ${a}_{2}$(ρ = 0.98).

These results suggest strong agreement between both methods, demonstrating that *eigenlenses* full-shape estimation from the pupil^16,17^ provides an accurate and consistent approximation of lens shape.

## Discussion

Most in vivo studies of the crystalline lens have relied on optical imaging techniques restricted to the central region visible through the pupil. In this study, we report the ability to visualize the equatorial region of the crystalline lens in vivo using OCT, achieved with mydriasis and off-axis (up to 45º) beam incidence. The equatorial region was successfully imaged in all eight subjects. Furthermore, we propose a method to merge on-axis and off-axis volumetric images to reconstruct the complete geometry of the crystalline lens.

Previous in vivo attempts to visualize the crystalline lens off-axis achieved a 17% increase in the visible areas of the lens^21^, by combining on-axis OCT images with four off-axis volumes. In the present work, we employed eight volumes acquired at large angular incidences, facilitated by an external stimulus, enabling direct visualization of the lens equator for the first time (Fig. 6). In addition, in contrast to prior literature^21^, we reconstructed the three-dimensional geometry for the different incidence angles and registered the entire data, providing complete sampling of the posterior surface and, consequently, the full 3-D geometry of the crystalline lens.

In earlier works^15–17^, we estimated the full shape of the crystalline lens from the regions of the anterior and posterior surfaces visible through the pupil. Using information of the full geometry of the crystalline lens that is accessible ex vivo, we developed mathematical models, so-called *eigenlenses*, to estimate the complete lens geometry based only on the visible portion. In the current work, by contrast, these parameters can be directly measured thanks to visualization of the entire crystalline lens. Although in some cases the full equator is not visible in all off-axis views, or the anterior corneal surface is partially obscured, preventing optical distortion correction of the subsequent surfaces and thus the use of that information in the reconstruction, the missing data are completed using *eigenlens* projections. Importantly, this does not involve estimating large unseen areas, as in our earlier estimation-based approaches, but rather interpolating small unrecovered portions. This distinction is clearly illustrated in Supplementary Fig. S5, which compares the geometry accessible through the pupil with that obtained using the proposed method.

Repeatability analysis revealed low coefficients of variation for all geometrical parameters in two subjects (CV < 1%), demonstrating excellent consistency across repeated measurements. We used the results using the proposed method as ground truth to evaluate the accuracy of estimation approaches^16,17^. Specifically, Bland-Altman and correlation plots have been used to compare the lens diameter, volume and lens surface area obtained with the proposed and with estimation methods (Fig. 7). Overall, the results demonstrate that the *eigenlens* estimation method provides an accurate approximation of the full crystalline lens parameters, making it a valuable alternative when peripheral visualization is not available and in clinical settings where rapid measurements are required.

We observed a strong positive correlation with age for all three quantified geometrical parameters, indicating age-related lens growth: diameter (ρ = 0.90, p < < 0.05), volume (ρ = 0.85, p < < 0.05), and surface area (ρ = 0.93, p < < 0.05). Although this was not the primary objective of the study and the sample size was limited (*n* = 8), the observed trends are consistent with previous findings obtained through estimation methods and other imaging techniques^38^.

It is important to perform optical distortion correction using three-dimensional ray-tracing methods, rather than the two-dimensional corrections typically implemented in commercial systems. Under on axis light incidence in a meridional scanning, the ray is refracted within the same plane as the OCT meridian, making 2-D correction sufficient. However, in cases of lateral light incidence, as in the present work, the ray is refracted outside the OCT meridian plane. In such conditions, 3-D correction is essential to achieve accurate results.

A potential source of error could be the lack of precision in the incidence angles for each off-axis measurement. However, acquiring images at various incidence angles provided some redundancy (i.e., information present in multiple angular measurements), alleviating the need to know the exact off-axis angle a priori (in addition, it can be determined a posteriori from the data). Nevertheless, once registered, this redundancy proved useful for verifying the accuracy of the registration process. The number and distribution of incidence angles could be further optimized to reduce acquisition time in clinical applications.

The influence of the gradient index of refraction (GRIN) of the crystalline lens on the reconstruction of the posterior surface was observed to be small when imaging the crystalline lens on axis^33,39^. However, the influence of the lens GRIN in the reconstruction of off-axis images was not studied. While high incidence rays not passing through the lens center may experience a different average refractive index, a limitation of the current study is that we assumed a constant refractive index. The lack of GRIN models that account for asymmetric lens surfaces prevents us from testing this hypothesis completely, but it is probable that only high incident rays will experience a highly different refractive index. The constant refractive index used was *n* = 1.417 for the crystalline lens. To assess the impact of this assumption, we reconstructed the posterior surface using three different refractive indices for the crystalline lens (*n* = 1.40, 1.417, and 1.44) for Subject # 6, covering the range of feasible values reported in the literature^33,34^. The DIA varied by approximately 0.4% (8.90 mm, 8.90 mm, and 8.86 mm, respectively), the VOL by 3% (142 mm³, 140 mm³, and 137 mm³), and the LSA by 2% (142 mm³, 140 mm³, and 137 mm³) across refractive indices. Thus, the influence of the elected refractive index can be considered small.

Finally, imaging subjects in both upright and prone position, a study found gravity-induced changes in the anterior chamber depth and lens thickness^40^. These changes were, on average, below 50 microns, and the eccentricities used in this study are well below 90º, so we expect this effect to be minimal in our measurements. Additionally, in some cases, the iris was not clearly visible in OCT images at very high incidence angles. Nevertheless, this affected less than 5% of the meridians and therefore did not impact the estimation of the iris plane and center, which are required for the registration process.

## Conclusion

In this study, three-dimensional reconstruction of the full crystalline lens shape from OCT images was achieved using lateral illumination incidence and a methodology to merge all measurements from the different angles. The results showed that quantified parameters of the crystalline lens (diameter, surface area, and volume) increased with age. Furthermore, the study demonstrated that the full lens shape estimated from the pupil region using *eigenlenses* closely matched the geometry obtained by registering multiple OCT views at different incidences. This agreement underscores the potential of eigenlens-based approaches to accurately reconstruct the full lens shape in clinical settings.

Enhanced visibility of the lens periphery, combined with the ability to fully quantify its geometry, may improve understanding and assessment of lens accommodation, contribute to studies of myopia progression and presbyopia onset, and support the design and customization of intraocular lens implants, ultimately enhancing visual outcomes and patient satisfaction in cataract and refractive surgeries.

## Supplementary Information

Below is the link to the electronic supplementary material.

Supplementary Material 1

Supplementary Material 2

Supplementary Material 3

Supplementary Material 4

Supplementary Material 5

Supplementary Material 6

Supplementary Material 7

## Data Availability

The datasets analysed during the current study are available in a GitHub repository: (https:/github.com/EduardoMartinezEnriquez/Complete-reconstruction-crystalline-lens-shape/tree/main).
