# Supplementary figures and images for: Reconstruction of the crystalline lens full-geometry from OCT images acquired with off-axis viewing
Source: Sci Rep. 2026 May 11;16:21474. doi: 10.1038/s41598-026-42539-3 (PMC13350928; doi:10.1038/s41598-026-42539-3)

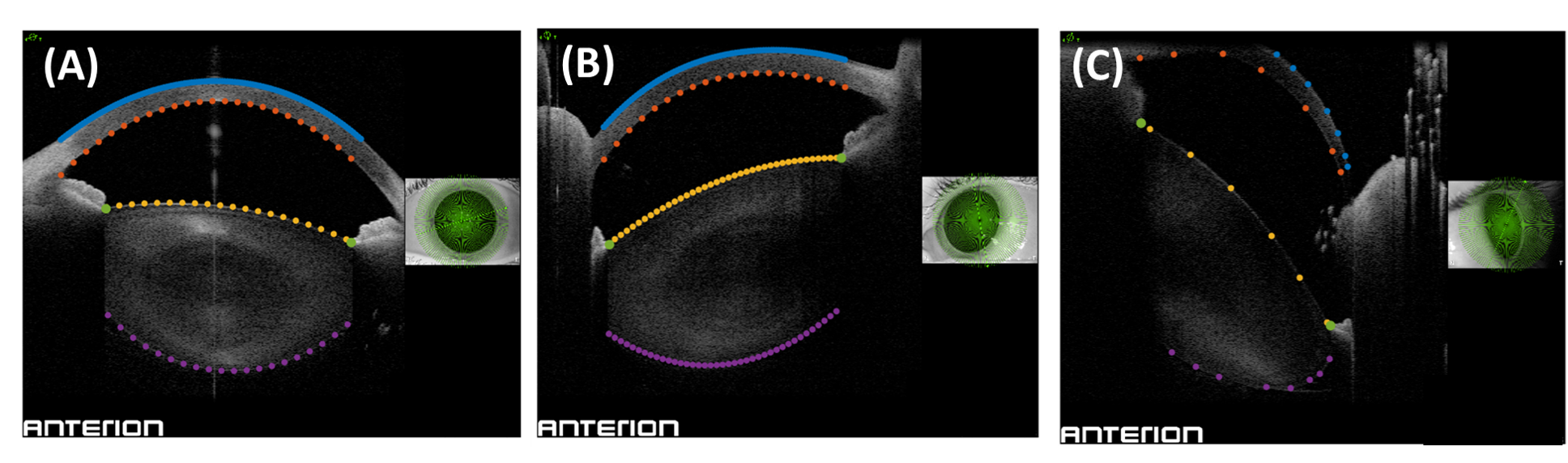

Supplement: Supplementary file 1 — Supplementary Material 1 [file 41598_2026_42539_MOESM1_ESM.png]

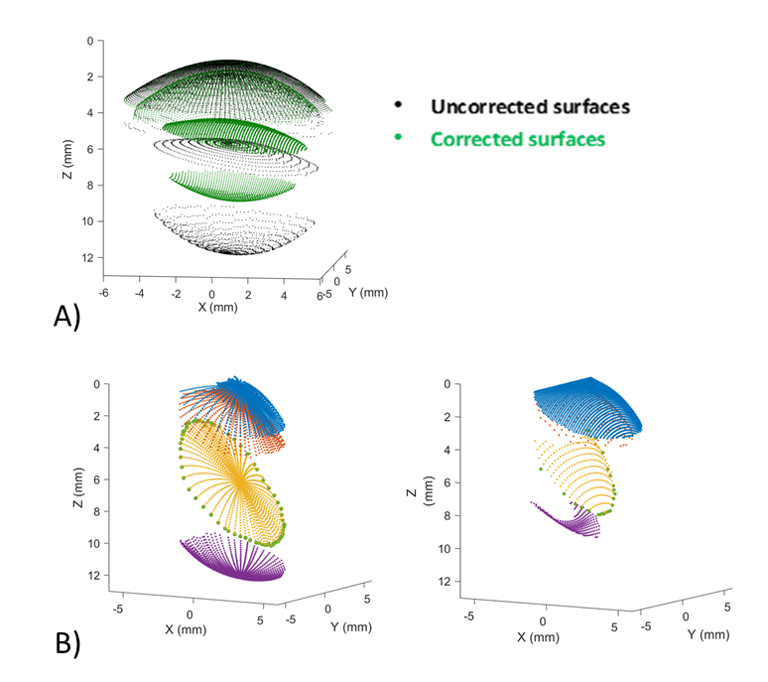

Supplement: Supplementary file 2 — Supplementary Material 2 [file 41598_2026_42539_MOESM2_ESM.png]

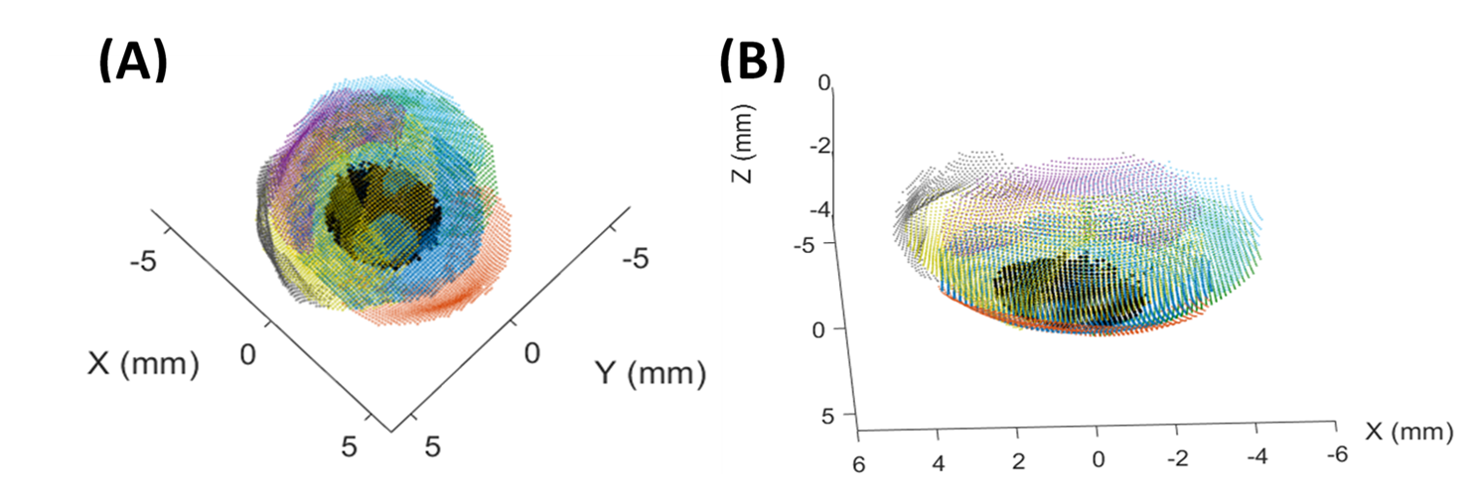

Supplement: Supplementary file 3 — Supplementary Material 3 [file 41598_2026_42539_MOESM3_ESM.png]

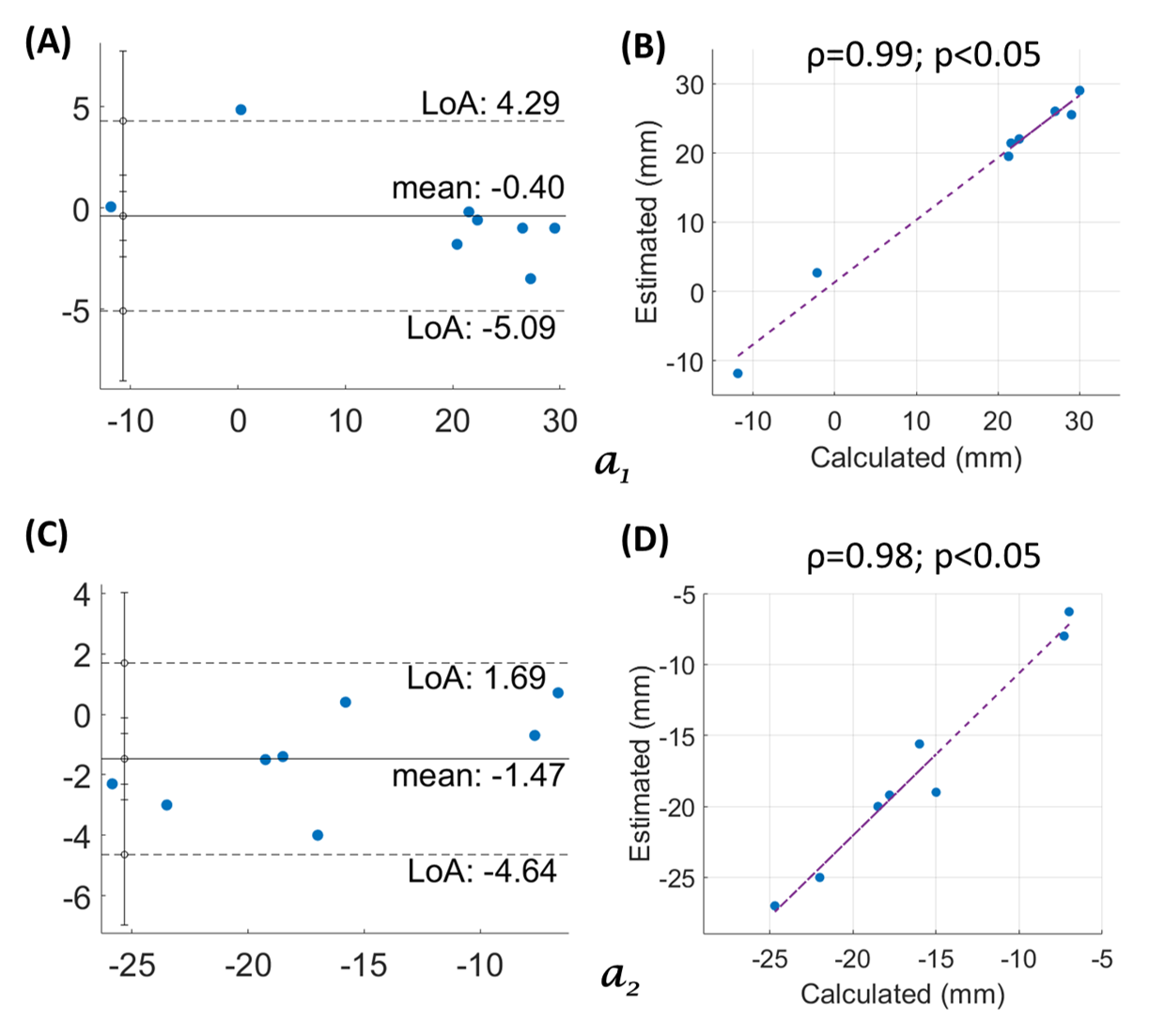

Supplement: Supplementary file 6 — Supplementary Material 6 [file 41598_2026_42539_MOESM6_ESM.png]
