# Supplementary material for: Reconstruction of the crystalline lens full-geometry from OCT images acquired with off-axis viewing
Source: Sci Rep. 2026 May 11;16:21474. doi: 10.1038/s41598-026-42539-3 (PMC13350928; doi:10.1038/s41598-026-42539-3)
Supplement: Supplementary file 7 — Supplementary Material 7 [file 41598_2026_42539_MOESM7_ESM.docx]

**Supplementary Figure S1.** Segmentation examples of B-scans for 3 different incidence angles (Subject #1, OS). (A) Normal incidence segmentation, B-scan # 27; (B) Nasal (30 degrees) incidence segmentation, B-scan # 60; (C) Temporal (45 degrees) incidence segmentation, B-scan # 8. Segmentation examples in (A) and (B) were performed with the automatic segmentation algorithm; (C) shows the points clicked manually with the manual algorithm. The blue color indicates anterior cornea surface; red, posterior cornea surface; yellow, anterior lens surface; purple, posterior lens surface; and green indicates the iris.

**Supplementary Figure S2.** Illustration of the optical distortion correction process. A) Normal incidence. Black: Uncorrected surfaces. Green: Corresponding corrected surfaces, OS. B) Temporal 30 degrees incidence before (left) and after optical distortion correction (right). Blue: anterior cornea surface; red: posterior cornea surface; yellow: anterior lens surface; purple: posterior lens surface; green: iris. Examples are for Subject #1 (OS).

**Supplementary Video S3.** Registration of the eight 3-D models from the measurements at different incidence angles.

**Supplementary Video S4**. Combination of data for eight incidences after the registration process.

**Supplementary Figure S5.** View of the central part (within the pupil) of the posterior surface of the crystalline lens usually obtained in clinical measurements (black) and the full shape of the posterior lens obtained with the proposed method (different colors). (A) Upper view; (B) lateral view. Subject # 1.

**Supplementary Figure S6.** Comparison between the proposed method using all orientations and the estimation methods that estimate the full shape of the lens from the limited central zone (pupil, on-axis data) using eigenlenses. (A) a_1_, Bland-Altman plot; (B) a_1_, correlation plot; (C) a_2_, Bland-Altman plot; (D) a_2_, correlation plot. In the correlation plots, “Calculated” (X-axis) refers to the values obtained with the proposed method, and “estimated” (Y-axis) refers to the values obtained with estimation methods from the pupil. Spearman correlation coefficient (ρ), p-values for testing the null hypothesis of no correlation (p), and best linear regression lines (purple dashed lines) are shown.
